# Spinal myoclonus post-CRT-D implantation: a rare case report of iodinated contrast neurotoxicity

**DOI:** 10.1093/ehjcr/ytag416

**Published:** 2026-06-10

**Authors:** Jaouad Nguadi, Mohamed Ennmer, Jihane Fagouri, Hicham Bouzelmat, Ali Chaib

**Affiliations:** Department of Cardiology, Mohammed V Military Training Hospital, Rabat 10000, Morocco; Department of Radiology, Mohammed V Military Training Hospital, Rabat 10000, Morocco; Department of Cardiology, Mohammed V Military Training Hospital, Rabat 10000, Morocco; Department of Cardiology, Mohammed V Military Training Hospital, Rabat 10000, Morocco; Department of Cardiology, Mohammed V Military Training Hospital, Rabat 10000, Morocco

**Keywords:** Cardiac resynchronization therapy, Heart failure, Case report, Iodinated contrast, Neurotoxicity, Spinal myoclonus, Magnetic resonance imaging

## Abstract

**Background:**

Contrast-induced neurotoxicity (CIN) is a rare complication of iodinated contrast agents, with spinal myoclonus representing an exceptionally uncommon manifestation (<0.01%). To date, no cases have been reported following cardiac resynchronization therapy with defibrillator (CRT-D) implantation.

**Case summary:**

A 65-year-old man with advanced heart failure (LVEF 28%, NYHA III, QRS 162 ms) underwent successful CRT-D implantation using 50 mL of iopamidol. Four hours post-procedure, he developed sudden, generalized, stimulus-sensitive spinal myoclonus with preserved consciousness. Electroencephalography was normal. Brain and cervical-thoracic spinal MRI on day 3 showed no ischaemic, haemorrhagic, or inflammatory lesions. Serum creatinine remained stable. Intravenous hydration and clonazepam resulted in complete resolution within 72 h, with no neurological sequelae at three-month follow-up (NYHA II, LVEF 38%).

**Discussion:**

This represents the first documented case of iodinated contrast-induced spinal myoclonus following CRT-D implantation, occurring with the lowest reported contrast volume (50 mL) and preserved renal function. Normal neuroimaging and rapid reversibility under supportive therapy confirm a functional, non-structural mechanism. This case expands the spectrum of CIN in device-based heart failure therapy and emphasizes the need for heightened clinical awareness during coronary sinus cannulation, even in low-risk patients. Early recognition and simple supportive measures ensure excellent outcomes.

Learning points

- Iodinated contrast can cause rare, fully reversible spinal myoclonus even with low volume (50 mL) and preserved renal function after CRT-D implantation.
- Normal brain and spinal MRI with rapid resolution under hydration and clonazepam supports a functional mechanism of contrast-induced neurotoxicity.
- Prompt recognition and supportive care in the post-procedural period ensure excellent neurological outcomes in this rare complication.

## Introduction

Cardiac resynchronization therapy with defibrillator (CRT-D) is a Class I, Level A recommendation for patients with systolic heart failure, reduced left ventricular ejection fraction (LVEF ≤35%), prolonged QRS duration (≥130 ms), and persistent symptoms despite optimal medical therapy, as outlined in the 2021 European Society of Cardiology guidelines.^1^ Fluoroscopy-guided cannulation of the coronary sinus necessitates the use of iodinated contrast agents, typically low-osmolar non-ionic compounds such as iopamidol, which are generally well-tolerated in patients with normal renal function.

Contrast-induced neurotoxicity (CIN), though rare with an incidence below 1% in cardiac procedures,^2^ may manifest as transient neurological symptoms including cortical blindness, encephalopathy, seizures, or, exceptionally, spinal myoclonus. Spinal myoclonus is characterized by rhythmic, involuntary, segmental muscle contractions originating from the spinal cord, without cortical correlates on electroencephalography (EEG). Fewer than 20 cases have been documented in the literature,^3^ all following coronary angiography or percutaneous coronary intervention. To date, no case has been reported following CRT-D implantation.

We describe the first documented instance of iodinated contrast-induced spinal myoclonus after CRT-D implantation, supported by a comprehensive diagnostic workup including normal brain and spinal MRI, and rapid, complete recovery under supportive management. This case broadens the recognized spectrum of CIN and highlights critical diagnostic and therapeutic considerations in device-based heart failure therapy.

## Summary figure

**Table:** 

Time point	Event	Key findings/Intervention
Pre-procedure 0.90 mg/dL	65 y/o ♂, idiopathic DCM	LVEF 28%, NYHA III, QRS 162 ms LBBB, eGFR 72 mL/min, Cr
t = 0 h lead	CRT-D implantation	50 mL iopamidol (370 mgI/mL), left subclavian access, quadripolar LV
	Fluoroscopy 14 min	Optimal lead parameters, no complication
t = 4 h	SYMPTOM ONSET	Generalized stimulus-sensitive spinal myoclonus, sudden agitation
	Rhythmic jerking limbs	GCS 15/15, hyperreflexia, no Babinski, stable vitals, Cr 0.88 mg/dL
t = 4–6 h 118 ms	Immediate diagnostics	EEG normal α 9–10 Hz, no epileptiform activity ECG BiV pacing, QRS
Day 3 (t = 72 h)	Brain + cervico-thoracic MRI (1.5 T)	T1/T2/FLAIR/DWI completely NORMAL
t = 0–72 h	TREATMENT	IV hydration 1.5 mL/kg/h + clonazepam 0.5 mg IV q6 h → 1 mg PO
		Complete resolution at 72 h
Day 5	Discharge	NYHA II, optimized GDMT + tailored CRT-D programming
3 months	Follow-up	LVEF ↑ 38%, no neurological sequelae

## Case presentation

A 65-year-old North African male with idiopathic dilated cardiomyopathy was referred for CRT-D implantation. He presented with New York Heart Association (NYHA) class III symptoms, LVEF of 28% by Simpson’s biplane method, and a QRS duration of 162 ms with left bundle branch block morphology in sinus rhythm. His medical history included hypertension managed with ramipril 5 mg daily, type 2 diabetes with HbA1c of 7.1% on metformin 1000 mg twice daily, and a 30-pack-year smoking history, which ceased five years prior. Renal function was preserved, with an estimated glomerular filtration rate of 72 mL/min/1.73 m^2^ and serum creatinine of 0.90 mg/dL. No drug allergies were reported.

The procedure was performed under local anaesthesia with 2% lidocaine and light sedation using 2 mg intravenous midazolam. Venous access was obtained via the left subclavian vein. A total of 50 mL of iopamidol (370 mgI/mL, low-osmolar, non-ionic) was administered in 5–10 mL boluses to visualize the coronary sinus ostium and branches. Leads were successfully positioned in the right atrial appendage, right ventricular apex, and a posterolateral branch of the coronary sinus using a quadripolar lead. Electrical parameters were optimal, with capture thresholds below 1.0 V at 0.4 ms, sensing amplitudes exceeding 8 mV, and impedances between 600 and 800 Ω. Fluoroscopy duration was 14 min, and no intraoperative complications occurred. Post-procedure chest X-ray confirmed correct lead positioning (*Figure 1*).

**Figure 1:**
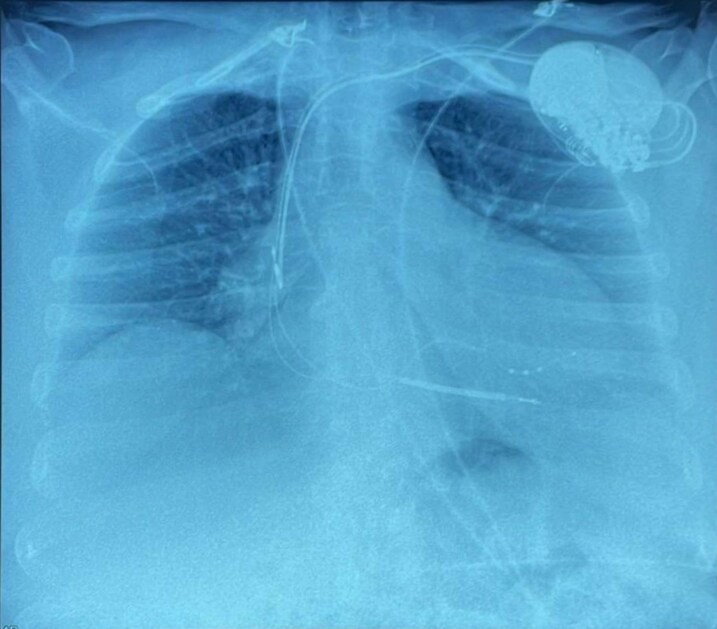
Posteroanterior chest X-ray 2 h post-CRT-D implantation. The right atrial lead is positioned in the appendage, the right ventricular lead at the apex, and the quadripolar coronary sinus lead in a posterolateral branch. No pneumothorax, haemothorax, or lead displacement is evident.

Four hours after the procedure, the patient developed sudden agitation accompanied by rhythmic, involuntary jerking movements involving flexion and extension of the upper and lower limbs, exacerbated by tactile or auditory stimuli. Neurological examination revealed a Glasgow Coma Scale of 15/15, no cranial nerve deficits, preserved muscle strength, symmetrical hyperreflexia without the Babinski sign, and no sensory level. Vital signs remained stable with blood pressure of 140/80 mmHg, heart rate of 78 bpm under biventricular pacing, and oxygen saturation of 96% on room air. Laboratory analysis showed mild troponin I elevation to 0.07 ng/mL (normal <0.04 ng/mL), normal electrolytes, and stable serum creatinine at 0.88 mg/dL. Electrocardiography demonstrated effective biventricular pacing with QRS narrowing to 118 ms.

Electroencephalography performed at symptom onset revealed a normal posterior dominant alpha rhythm of 9–10 Hz without epileptiform discharges, slowing, or asymmetry (*Figure 2*). Due to the initial unavailability of magnetic resonance imaging (MRI), brain and cervical-thoracic spinal MRI were conducted on day 3 using a 1.5 Tesla scanner. Sequences included T1, T2, FLAIR, and diffusion-weighted imaging, all of which demonstrated no acute ischaemic, haemorrhagic, or inflammatory lesions and no abnormal contrast enhancement within the spinal cord (*Figure 3*). Serum creatinine remained stable at 0.87 mg/dL on day 2 and 0.85 mg/dL on day 3, confirming preserved renal clearance. Lumbar puncture was not performed, given the absence of meningism and rapid clinical improvement.

**Figure 2:**
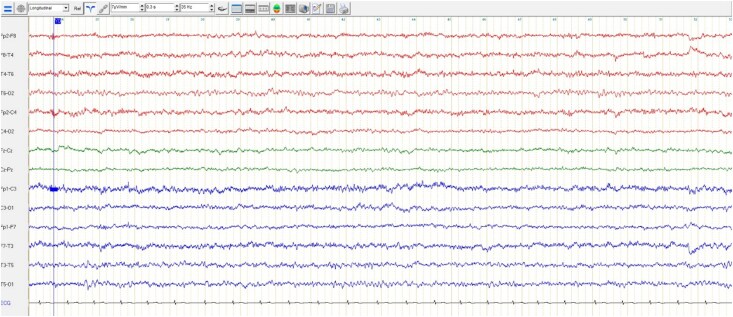
Eighteen-channel electroencephalography recorded at symptom onset (4 h post-procedure). Normal posterior dominant alpha rhythm of 9–10 Hz is observed, with no epileptiform discharges, focal slowing, or asymmetry. Calibration: 7 µV/mm, time base 15 mm/s.

**Figure 3:**
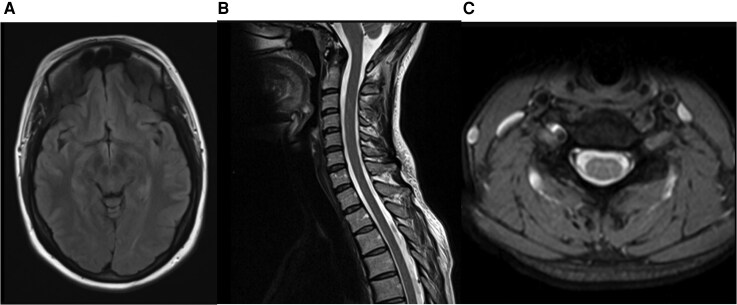
Brain and cervical-thoracic spinal magnetic resonance imaging performed on day 3 (1.5 Tesla). (*A*) Axial FLAIR sequence of the brain shows no parenchymal abnormality. (*B*) Sagittal T2-weighted image of the spinal cord demonstrates normal calibre and signal intensity. (*C*) Axial T2-weighted image at T6 level reveals no cord oedema or abnormal enhancement. Overall, the central nervous system is structurally normal.

Following multidisciplinary consultation involving cardiology, neurology, and intensive care specialists, a diagnosis of iodinated contrast-induced spinal myoclonus was established. Treatment was initiated promptly with intravenous hydration using 2 L of 5% dextrose over 4 h, followed by 1.5 mL/kg/h, to enhance contrast elimination. Clonazepam was administered at 0.5 mg intravenously every 6 h for 48 h, then transitioned to 1 mg orally daily. The patient was monitored in the intensive care unit with daily neurological assessments using the Richmond Agitation-Sedation Scale, targeting a score below zero.

Myoclonus significantly diminished by 48 h, manifesting only as occasional limb twitches, and resolved completely by 72 h. The patient was discharged on day 5 with optimized heart failure therapy, including sacubitril/valsartan 49/51 mg twice daily, ivabradine 5 mg twice daily, bisoprolol 5 mg daily, and spironolactone 25 mg daily, alongside tailored CRT-D programming. At three-month follow-up, the patient reported NYHA class II symptoms, LVEF had improved to 38%, and no neurological sequelae were observed. Informed written consent was obtained from the patient for publication of this case report and accompanying images.

## Discussion

Iodinated contrast agents are associated with neurological complications in fewer than 1% of intravascular procedures.^2^ CIN typically manifests within 24 to 48 h and is thought to result from osmotic disruption of the blood-brain or blood-spinal cord barrier, direct endothelial toxicity, or secondary inflammatory cascades. Spinal myoclonus, an exceptionally rare presentation with an estimated incidence below 0.01% following cardiac interventions,^3^ likely arises from transient hyperexcitability of anterior horn cells, possibly mediated by contrast extravasation into the spinal cord via osmotic opening or active transport mechanisms. Animal studies have demonstrated reversible spinal cord signal changes with high-dose iopamidol, though such findings are absent at clinical doses.

Risk factors for CIN include advanced age, heart failure, renal impairment, and high contrast volume; notably, none were prominently present in this case beyond age and heart failure. The rapid onset at 4 h, lowest reported contrast volume of 50 mL, and occurrence following CRT-D implantation distinguish this case from previously published reports, all of which followed coronary angiography or percutaneous coronary intervention^4–6^ (*Table 1*).

**Table 1:** Comparison of published cases of contrast-induced spinal myoclonus

Reference	Age/Sex	Procedure	Contrast agent	Volume (mL)	Onset (h)	MRI Findings	EEG	Treatment	Resolution
Gürses *et al*.^4^	77/M	PCI	Iohexol	150	6	Normal	Normal	Diazepam	3 days
Al-Hwiesh *et al*.^5^	68/F	Coronary angiography	Iopamidol	120	12	Normal	Normal	Clonazepam	48 h
Li *et al*.^6^	72/M	Carotid stenting	Iomeprol	80	8	Normal	Not reported	Hydration	4 days
Present case	65/M	CRT-D	Iopamidol	50	4	Normal (brain + spine)	Normal	Hydration + clonazepam	72 h

The diagnostic approach relied on clinical features—rhythmic, stimulus-sensitive jerking with preserved consciousness—and was supported by normal EEG, excluding cortical seizures, and normal brain and spinal MRI, ruling out structural pathology. The rapid resolution with hydration and clonazepam further corroborated the toxic, reversible nature of the condition. Differential diagnoses such as metabolic encephalopathy, ischaemic myelopathy, or drug-induced myoclonus were excluded based on stable biochemistry, imaging, and clinical course.^5^

Management remains supportive in the absence of formal guidelines. Intravenous hydration at 1–2 mL/kg/h accelerates contrast clearance, while benzodiazepines such as clonazepam enhance GABA-A-mediated inhibition, suppressing spinal hyperexcitability. Levetiracetam may be considered in refractory cases. Neuroimaging, when feasible, is essential to exclude alternative aetiologies, though normal findings, as in this case, are consistent with the functional mechanism of contrast-induced spinal myoclonus.^6^

Preventive strategies include the use of low-osmolar non-ionic agents, minimization of contrast volume below 100 mL, and consideration of pre-procedure hydration or corticosteroid-antihistamine prophylaxis in high-risk patients. This case underscores the need for clinical vigilance even in low-risk scenarios and highlights the value of multidisciplinary collaboration in achieving rapid diagnosis and recovery.^4–6^

## Conclusion

Spinal myoclonus following CRT-D implantation represents a rare but fully reversible complication of iodinated contrast neurotoxicity. This first reported case, occurring with only 50 mL of iopamidol and normal renal function, demonstrates that even minimal contrast exposure can precipitate transient spinal cord dysfunction in susceptible individuals. Normal brain and spinal MRI, stable renal parameters, and complete resolution within 72 h under hydration and clonazepam therapy affirm the functional, non-structural basis of this phenomenon.

Clinicians should suspect spinal myoclonus in any patient presenting with rhythmic, stimulus-sensitive jerking within 24 h of contrast administration, irrespective of procedure type or renal function. Early recognition and simple supportive measures prevent unnecessary investigations and ensure excellent outcomes. This case expands the recognized spectrum of CIN and reinforces the importance of heightened awareness during device implantation procedures.

## Data Availability

The data underlying this article are available in the article and in its online supplementary material.

## Lead author biography

I am Jaouad Nguadi, a 33-year-old cardiac electrophysiologist from Morocco. I completed my fellowship in cardiac rhythmology in both Morocco and France, training at Clinique Les Fontaines, Clinique Anthony, and Hôpital Privé Jacques Cartier (Massy, France). I obtained my DIU in Rhythmology and Cardiac Stimulation from Université Paris Cité, France.

## Notes

**Consent:** The authors confirm that written informed consent for publication of this case report and accompanying images was obtained from the patient.
